# Dimethyl­ammonium bis­(3-oxidonaphthalene-2-carboxyl­ato)borate hemihydrate

**DOI:** 10.1107/S1600536807066810

**Published:** 2007-12-18

**Authors:** Mustafa Tombul, Kutalmış Güven, Ingrid Svoboda

**Affiliations:** a Department of Chemistry, Faculty of Arts and Sciences, University of Kırıkkale, Campus, Yahşihan, 71450 Kırıkkale, Turkey; b Department of Physics, Faculty of Arts and Sciences, University of Kırıkkale, Campus, Yahşihan, 71450 Kırıkkale, Turkey; c Structural Research, Material Science, Darmstadt University of Technology, Petersen Strasse 23, D-64287 Darmstadt, Germany

## Abstract

The title compound, C_2_H_8_N^+^·C_22_H_12_BO_6_^−^·0.5H_2_O, was synthesized under atmospheric conditions in the presence of dimethyl­formamide acting as a template. The structure is composed of [NH_2_(CH_3_)_2_]^+^ cations, bis­(3-oxidonaphthalene-2-carboxyl­ato)borate anions and water mol­ecules. The water molecule lies on a twofold rotation axis. The stabilization of the crystal structure comes from electrostatic inter­actions and is assisted by inter­molecular O—H⋯O and N—H⋯O hydrogen bonds between the layers.

## Related literature

For related literature, see: Carr *et al.* (2005 ▶); Downard *et al.* (2002 ▶); Errington *et al.* (1999 ▶); Green *et al.* (2000 ▶); Grice *et al.* (1999 ▶); Li & Liu (2006 ▶); Schubert *et al.* (2000 ▶); Tombul *et al.* (2003 ▶); Tombul, Guven, Büyükgüngör *et al.* (2007 ▶); Touboul *et al.* (2003 ▶); Zhang & Liu (2006 ▶); Zhang *et al.* (2005 ▶).
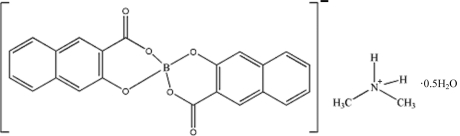


## Experimental

### 

#### Crystal data

C_2_H_8_N^+^·C_22_H_12_BO_6_^−^·0.5H_2_O
*M* *_r_* = 438.24
Monoclinic, 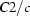

*a* = 32.011 (3) Å
*b* = 9.774 (1) Å
*c* = 14.742 (1) Å
β = 112.628 (7)°
*V* = 4257.2 (7) Å^3^
*Z* = 8
Mo *K*α radiation
μ = 0.10 mm^−1^
*T* = 303 (2) K
0.48 × 0.08 × 0.08 mm

#### Data collection

Oxford Diffraction Xcalibur diffractometer with a Sapphire CCD detector
Absorption correction: numerical [using a multifaceted crystal model based on expressions derived (Clark & Reid, 1995 ▶)] *T*_min_ = 0.981, *T*_max_ = 0.994
16173 measured reflections
4321 independent reflections
1708 reflections with *I* > 2σ(*I*)
*R*_int_ = 0.079

#### Refinement

*R*[*F* ^2^ > 2σ(*F* ^2^)] = 0.093
*wR*(*F* ^2^) = 0.152
*S* = 1.12
4321 reflections
299 parameters
3 restraints
H atoms treated by a mixture of independent and constrained refinement
Δρ_max_ = 0.21 e Å^−3^
Δρ_min_ = −0.23 e Å^−3^

### 

Data collection: *CrysAlis CCD* (Oxford Diffraction, 2006 ▶); cell refinement: *CrysAlis RED* (Oxford Diffraction, 2006 ▶); data reduction: *CrysAlis RED*; program(s) used to solve structure: *SHELXS97* (Sheldrick, 1997 ▶); program(s) used to refine structure: *SHELXL97* (Sheldrick, 1997 ▶); molecular graphics: *Mercury* (Macrae *et al.*, 2006 ▶); software used to prepare material for publication: *publCIF* (Westrip, 2008 ▶).

## Supplementary Material

Crystal structure: contains datablocks global, I. DOI: 10.1107/S1600536807066810/at2517sup1.cif

Structure factors: contains datablocks I. DOI: 10.1107/S1600536807066810/at2517Isup2.hkl

Additional supplementary materials:  crystallographic information; 3D view; checkCIF report

## Figures and Tables

**Table:** 

O1—B1	1.427 (5)
O2—B1	1.496 (5)
O4—B1	1.426 (5)
O5—B1	1.502 (5)

**Table:** 

O4—B1—O1	110.7 (4)
O4—B1—O2	107.7 (3)
O1—B1—O2	112.4 (3)
O4—B1—O5	112.0 (3)
O1—B1—O5	107.9 (3)
O2—B1—O5	106.1 (4)

**Table 2:** Hydrogen-bond geometry (Å, °)

*D*—H⋯*A*	*D*—H	H⋯*A*	*D*⋯*A*	*D*—H⋯*A*
N5—H25*A*⋯O3^i^	1.00	1.88	2.849 (4)	162
N5—H25*B*⋯O6^ii^	0.95	2.12	2.839 (4)	131
N5—H25*B*⋯O3^iii^	0.95	2.33	2.871 (4)	116
O7—H27⋯O5	0.85 (3)	2.18 (4)	3.010 (3)	163.3 (12)

Symmetry codes: (i) 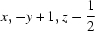; (ii) 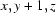; (iii) 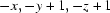.

**Fig. 1.**
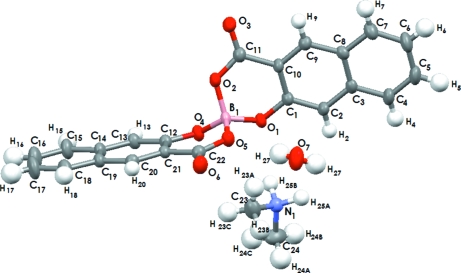
Showing the atom-labelling scheme of (I). Displacement ellipsoids are drawn at the 50% probability level.

**Fig. 2.**
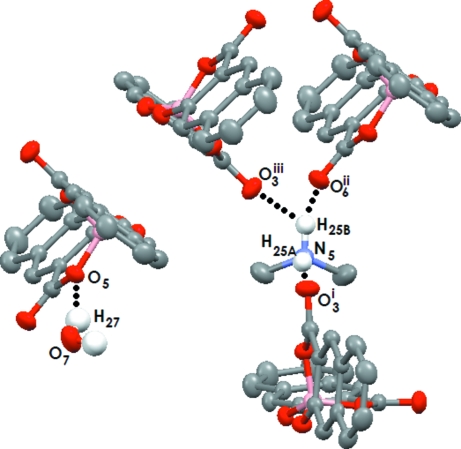
Hydrogen bond labelling scheme for (I). Dashed lines indicate hydrogen bonds. Some hydrogen atoms were omited for clarity). [Symmetry codes: (i): x, -y + 1, z - 1/2, (ii): x, y + 1, z, (iii):-x, -y + 1, -z + 1].

## supplementary crystallographic information

### Comment

Owing to their rich structural chemistry and potential applications in mineralogy (Grice *et al.*, 1999) and nonlinear optical materials (Touboul *et al.*, 2003), borates have provided an abounding area of research for over half a century. Boron can form a large variety of compounds due to the complexity of the structures involved. Boron atom coordinate with oxygen not only in fourfold coordination (tetrahedral, BO_4_) but also in threefold coordination (triangular, BO_3_). These BO_3_ and BO_4_ groups favour polymerization *via* common corners into large polynuclear anion units including isolated or finite clusters, chains, sheets, and networks (Grice *et al.*, 1999). Most borates synthesized and studied to date have been prepared under the templating effect of inorganic cations, such as alkali-metal, alkaline-earth, rare-earth or transition cations. Accordingly, many borate systems taking into account alkali metal, alkaline earth metal, rare-earth, and transition metals have been explored in recent years (Schubert *et al.*, 2000). Currently, studies conducted in this area are primarily focused on improving existing materials utilized and search for new materials. In comparison to inorganic borates, synthesis, crystal structure and application of organic borates seem to be insufficient (Li & Liu, 2006; Zhang & Liu, 2006; Carr *et al.*, 2005; Zhang *et al.*, 2005; Downard *et al.*, 2002; Green *et al.*, 2000). As part of our ongoing study of the synthesis and structure of organoborate complexes (Errington *et al.*, 1999; Tombul *et al.*, 2003; Tombul, Guven, Büyükgüngör *et al.*, 2007), we have prepared a new organically templated borate, (I), using dimethylformamide as the structure-directing agent.

The asymmetric unit of (I) (Fig.1) consists of [BO_4_(C_11_H_6_O)_2_]^-^ anions, [NH_2_(CH_3_)_2_]^+^ cations and water molecule. The [BO_4_(C_11_H_6_O)_2_]^-^ anion consists of one set of distorted [BO_4_] tetrahedra and two sets of slightly deformed [C_11_H_6_O] planes with oxygen atoms as sharing vertexes. The boron atom is bonded to four oxygen atoms to form a highly tetrahedral environment (mean OBO bond angle of 109.46 (3)°). The B—O bond lengths are typical for such tetrahedral borates, with *B*—O_carboxyl_ bond lengths (mean 1.499 (5) Å) being slightly longer than B—O_hyroxyl_ bond lengths (mean 1.427 (5) Å). Each [C_11_H_6_O] unit is almost planar, and both [C_11_H_6_O] units in BO_4_(C_11_H_6_O)_2_]^-^ are nearly perpendicular to each other. The borate rings highly coplanar with the corresponding aryl rings [with 0.0779 Å r.m.s. deviation for Plane A (C1—C11/O1—O3) and 0.0648 Å r.m.s. deviation for Plane B (C12—C22/,O4—O6)]. The coordination planes (Planes C (O2, B1, O1) and D (O5, B1, O4) intersects at an angle of 89.73 (26) °. Dimethylammonium cation is distorted tetrahedral environment with CNC bond angle of 113.5 (4) ° and located with a large distance from the anion (B—N distance 6.108 (15) Å).

In the crystal structure [BO_4_(C_11_H_6_O)_2_]^-^ anions and [NH_2_(CH_3_)_2_]^+^ cations are discrete units and they interact both electrostatically and *via* N—H···O hydrogen bonds with N···O distances in the range 2.839 (4) Å – 2.872 (4) Å (Fig.2). The water molecule is also involved in normal, slightly bent, hydrogen bond with the borate anion at a distance of 3.012 (3) Å. The acceptors are all carboxylate O atoms of the aromatic ring (Table 2). For the synthesis of (I), dimethylformamide acts not only as the solvent, but also as the reactant and it decomposes under experimental conditions forming [NH_2_(CH_3_)_2_]^+^ cations.

### Experimental

For the preparation of title compound, (I), B(OH)_3 _(133 mg, 2.16 mmol,) was carefully added to a stirred DMF (10.0 ml) solution of 3-hydroxynaphthalene-2-carboxylic acid (875 mg, 4.65 mmol) at ambient temperature. The reaction mixture initially gave a brown solution which was stirred at 398 K for 2.5 h until all became a gel-like material. This product was then redissolved in the mixture of MeOH/CH_2_Cl_2_ (10 ml; 1:1) and allowed to stand at room temperature for a couple of hours, whereupon transparent and fine crystals were harvested. Yield, 79% (based on B(OH)_3_); Elemental analysis: (Found): C 65.48, H 5.18, N 3.19%. Calculated for C_24_H_22_O_7_NB: C 64.45, H 4.96, N 3.13%. ^1^H NMR (d^6^-DMSO-CDCl_3_, 298 K, TMS): δ (p.p.m.): All aromatic signals are observed between the region at 7.12 and 8.43. ^13^C NMR (d^6^-DMSO-CDCl_3_, 298 K, TMS): δ (p.p.m.): δ 119.7, 122.5, 128.1, 132.2, 142.4, 160.0, 161.6, 169.7, 177.0.

### Refinement

The H27 atom was located in a difference map and refined freely. Other H atoms were positioned geometrically and refined using a riding model, with C—H = 0.93–0.96 Å and N—H = 0.9530–1.0035 Å with *U*_iso_(H) = 1.2 or 1.5 times *U*_eq_(C,*N*) of the parent atom.

### Crystal data

**Table:**

C_2_H_8_N^+^·C_22_H_12_BO_6_^–^·0.5H_2_O	*F*_000_ = 1832
*M**_r_* = 438.24	*D*_x_ = 1.367 Mg m^−^^3^
Monoclinic, *C*2/*c*	Mo *K*α radiation λ = 0.71073 Å
Hall symbol: -C 2yc	Cell parameters from 2075 reflections
*a* = 32.011 (3) Å	θ = 2.5–21.1º
*b* = 9.774 (1) Å	µ = 0.10 mm^−^^1^
*c* = 14.742 (1) Å	*T* = 303 (2) K
β = 112.628 (7)º	Rod shape, light yellow
*V* = 4257.2 (7) Å^3^	0.48 × 0.08 × 0.08 mm
*Z* = 8	

### Data collection

**Table:**

Oxford Diffraction Xcalibur diffractometer with a Sapphire CCD detector	4321 independent reflections
Radiation source: fine-focus sealed tube	1708 reflections with *I* > 2σ(*I*)
Monochromator: graphite	*R*_int_ = 0.079
Detector resolution: 8.4012 pixels mm^-1^	θ_max_ = 26.4º
*T* = 303(2) K	θ_min_ = 2.5º
Rotation method data acquisition using ω and φ scans	*h* = −40→40
Absorption correction: numerical[absorption correction using a multifaceted crystal model based on expressions derived (Clark & Reid, 1995)]	*k* = −9→12
*T*_min_ = 0.981, *T*_max_ = 0.994	*l* = −18→18
16173 measured reflections	

### Refinement

**Table:**

Refinement on *F*^2^	Secondary atom site location: difference Fourier map
Least-squares matrix: full	Hydrogen site location: inferred from neighbouring sites
*R*[*F*^2^ > 2σ(*F*^2^)] = 0.093	H atoms treated by a mixture of independent and constrained refinement
*wR*(*F*^2^) = 0.152	*w* = 1/[σ^2^(*F*_o_^2^) + (0.0247*P*)^2^ + 7.7951*P*] where *P* = (*F*_o_^2^ + 2*F*_c_^2^)/3
*S* = 1.12	(Δ/σ)_max_ < 0.001
4321 reflections	Δρ_max_ = 0.21 e Å^−^^3^
299 parameters	Δρ_min_ = −0.23 e Å^−^^3^
3 restraints	Extinction correction: none
Primary atom site location: structure-invariant direct methods	

### Special details

**Table:**

Geometry. All e.s.d.'s (except the e.s.d. in the dihedral angle between two l.s. planes) are estimated using the full covariance matrix. The cell e.s.d.'s are taken into account individually in the estimation of e.s.d.'s in distances, angles and torsion angles; correlations between e.s.d.'s in cell parameters are only used when they are defined by crystal symmetry. An approximate (isotropic) treatment of cell e.s.d.'s is used for estimating e.s.d.'s involving l.s. planes.
Refinement. Refinement of *F*^2^ against ALL reflections. The weighted *R*-factor *wR* and goodness of fit *S* are based on *F*^2^, conventional *R*-factors *R* are based on *F*, with *F* set to zero for negative *F*^2^. The threshold expression of *F*^2^ > σ(*F*^2^) is used only for calculating *R*-factors(gt) *etc*. and is not relevant to the choice of reflections for refinement. *R*-factors based on *F*^2^ are statistically about twice as large as those based on *F*, and *R*- factors based on ALL data will be even larger.

### Fractional atomic coordinates and isotropic or equivalent isotropic displacement parameters (Å^2^)

**Table:**

	*x*	*y*	*z*	*U*_iso_*/*U*_eq_	
O1	−0.01987 (8)	0.4067 (3)	0.37753 (18)	0.0487 (8)	
O2	−0.03911 (9)	0.2873 (3)	0.5007 (2)	0.0520 (8)	
O3	0.01354 (9)	0.2159 (3)	0.6392 (2)	0.0643 (9)	
O4	−0.09425 (8)	0.4084 (3)	0.37123 (19)	0.0474 (7)	
O5	−0.06167 (9)	0.2000 (3)	0.33692 (19)	0.0498 (8)	
O6	−0.10420 (9)	0.0190 (3)	0.2734 (2)	0.0608 (9)	
C1	0.02357 (13)	0.3624 (4)	0.4222 (3)	0.0421 (11)	
C2	0.05411 (13)	0.3882 (4)	0.3807 (3)	0.0443 (11)	
H2	0.0451	0.4358	0.3216	0.053*	
C3	0.09922 (13)	0.3435 (4)	0.4264 (3)	0.0423 (11)	
C4	0.13136 (14)	0.3649 (5)	0.3838 (3)	0.0564 (13)	
H4	0.1230	0.4126	0.3249	0.068*	
C5	0.17420 (16)	0.3169 (5)	0.4274 (4)	0.0693 (15)	
H5	0.1947	0.3314	0.3976	0.083*	
C6	0.18799 (15)	0.2461 (5)	0.5163 (4)	0.0712 (15)	
H6	0.2175	0.2135	0.5452	0.085*	
C7	0.15843 (14)	0.2246 (5)	0.5608 (3)	0.0615 (13)	
H7	0.1679	0.1784	0.6205	0.074*	
C8	0.11340 (13)	0.2721 (4)	0.5170 (3)	0.0443 (11)	
C9	0.08061 (13)	0.2483 (4)	0.5579 (3)	0.0457 (11)	
H9	0.0890	0.2026	0.6176	0.055*	
C10	0.03693 (13)	0.2910 (4)	0.5115 (3)	0.0398 (10)	
C11	0.00351 (14)	0.2617 (4)	0.5560 (4)	0.0482 (12)	
C12	−0.13259 (13)	0.3378 (4)	0.3605 (3)	0.0406 (10)	
C13	−0.16686 (12)	0.4009 (4)	0.3771 (3)	0.0462 (11)	
H13	−0.1638	0.4923	0.3962	0.055*	
C14	−0.20682 (13)	0.3303 (5)	0.3661 (3)	0.0470 (12)	
C15	−0.24235 (14)	0.3923 (5)	0.3855 (3)	0.0686 (15)	
H15	−0.2397	0.4828	0.4065	0.082*	
C16	−0.28029 (16)	0.3202 (6)	0.3738 (4)	0.0833 (17)	
H16	−0.3035	0.3628	0.3863	0.100*	
C17	−0.28545 (16)	0.1834 (6)	0.3434 (4)	0.0858 (17)	
H17	−0.3117	0.1356	0.3362	0.103*	
C18	−0.25178 (14)	0.1214 (5)	0.3245 (3)	0.0694 (14)	
H18	−0.2551	0.0304	0.3046	0.083*	
C19	−0.21161 (13)	0.1922 (5)	0.3345 (3)	0.0457 (11)	
C20	−0.17613 (13)	0.1312 (4)	0.3159 (3)	0.0463 (11)	
H20	−0.1792	0.0412	0.2937	0.056*	
C21	−0.13709 (12)	0.2008 (4)	0.3295 (3)	0.0379 (10)	
C22	−0.10027 (14)	0.1318 (5)	0.3107 (3)	0.0466 (11)	
B1	−0.05381 (15)	0.3300 (5)	0.3956 (4)	0.0444 (13)	
N5	−0.06832 (10)	0.8557 (4)	0.1613 (2)	0.0518 (9)	
H25A	−0.0368	0.8299	0.1688	0.062*	
H25B	−0.0619	0.9008	0.2225	0.062*	
C23	−0.09329 (15)	0.7284 (5)	0.1570 (4)	0.0791 (16)	
H23A	−0.0763	0.6713	0.2117	0.095*	
H23B	−0.0979	0.6813	0.0967	0.095*	
H23C	−0.1221	0.7494	0.1596	0.095*	
C24	−0.09181 (16)	0.9503 (5)	0.0806 (3)	0.0898 (18)	
H24A	−0.0997	0.9036	0.0190	0.108*	
H24B	−0.0723	1.0261	0.0831	0.108*	
H24C	−0.1188	0.9833	0.0869	0.108*	
O7	0.0000	0.0715 (4)	0.2500	0.0849 (15)	
H27	−0.0156 (12)	0.1236 (14)	0.271 (3)	0.102*	

### Atomic displacement parameters (Å^2^)

**Table:**

	*U*^11^	*U*^22^	*U*^33^	*U*^12^	*U*^13^	*U*^23^
O1	0.0322 (16)	0.061 (2)	0.0533 (19)	−0.0011 (15)	0.0168 (15)	0.0074 (15)
O2	0.0364 (16)	0.071 (2)	0.053 (2)	−0.0023 (16)	0.0217 (15)	0.0056 (17)
O3	0.0504 (18)	0.088 (3)	0.058 (2)	−0.0010 (17)	0.0247 (17)	0.0188 (19)
O4	0.0370 (16)	0.0427 (19)	0.0651 (19)	−0.0031 (14)	0.0226 (15)	−0.0049 (15)
O5	0.0442 (17)	0.047 (2)	0.063 (2)	0.0006 (15)	0.0262 (16)	−0.0089 (16)
O6	0.0632 (19)	0.051 (2)	0.078 (2)	0.0015 (17)	0.0375 (17)	−0.0150 (18)
C1	0.037 (3)	0.042 (3)	0.048 (3)	−0.006 (2)	0.017 (2)	0.000 (2)
C2	0.040 (3)	0.050 (3)	0.045 (3)	−0.008 (2)	0.020 (2)	0.000 (2)
C3	0.038 (3)	0.043 (3)	0.049 (3)	−0.010 (2)	0.019 (2)	−0.012 (2)
C4	0.046 (3)	0.067 (3)	0.067 (3)	−0.013 (3)	0.033 (3)	−0.011 (3)
C5	0.052 (3)	0.084 (4)	0.088 (4)	−0.009 (3)	0.045 (3)	−0.012 (3)
C6	0.040 (3)	0.076 (4)	0.103 (4)	0.001 (3)	0.033 (3)	0.001 (3)
C7	0.043 (3)	0.060 (3)	0.079 (3)	0.003 (2)	0.020 (3)	0.007 (3)
C8	0.038 (3)	0.042 (3)	0.049 (3)	−0.004 (2)	0.012 (2)	−0.003 (2)
C9	0.046 (3)	0.045 (3)	0.049 (3)	−0.003 (2)	0.021 (2)	0.000 (2)
C10	0.035 (2)	0.040 (3)	0.048 (3)	−0.005 (2)	0.021 (2)	0.001 (2)
C11	0.046 (3)	0.047 (3)	0.056 (3)	−0.004 (2)	0.024 (3)	−0.002 (3)
C12	0.038 (2)	0.038 (3)	0.047 (3)	−0.001 (2)	0.017 (2)	−0.002 (2)
C13	0.040 (2)	0.041 (3)	0.060 (3)	0.001 (2)	0.021 (2)	−0.008 (2)
C14	0.034 (2)	0.054 (3)	0.056 (3)	0.005 (2)	0.020 (2)	−0.003 (2)
C15	0.047 (3)	0.069 (4)	0.100 (4)	0.002 (3)	0.039 (3)	−0.007 (3)
C16	0.054 (3)	0.085 (5)	0.123 (5)	0.004 (3)	0.048 (3)	−0.006 (4)
C17	0.049 (3)	0.086 (5)	0.133 (5)	−0.015 (3)	0.046 (3)	−0.006 (4)
C18	0.053 (3)	0.070 (4)	0.089 (4)	−0.011 (3)	0.031 (3)	−0.004 (3)
C19	0.035 (2)	0.053 (3)	0.050 (3)	−0.003 (2)	0.018 (2)	0.001 (2)
C20	0.048 (3)	0.045 (3)	0.047 (3)	−0.002 (2)	0.019 (2)	−0.005 (2)
C21	0.033 (2)	0.041 (3)	0.040 (3)	0.004 (2)	0.014 (2)	0.001 (2)
C22	0.049 (3)	0.045 (3)	0.048 (3)	0.001 (3)	0.021 (2)	−0.002 (2)
B1	0.027 (3)	0.057 (4)	0.050 (3)	−0.002 (3)	0.017 (3)	0.001 (3)
N5	0.048 (2)	0.056 (2)	0.052 (2)	0.006 (2)	0.0200 (19)	−0.003 (2)
C23	0.069 (3)	0.074 (4)	0.097 (4)	−0.020 (3)	0.035 (3)	−0.026 (3)
C24	0.106 (4)	0.096 (5)	0.065 (4)	0.040 (4)	0.029 (3)	0.013 (3)
O7	0.086 (4)	0.060 (3)	0.135 (4)	0.000	0.071 (3)	0.000

### Geometric parameters (Å, °)

**Table:**

O1—C1	1.360 (4)	C12—C21	1.405 (5)
O1—B1	1.427 (5)	C13—C14	1.406 (5)
O2—C11	1.316 (4)	C13—H13	0.9300
O2—B1	1.496 (5)	C14—C15	1.412 (5)
O3—C11	1.227 (4)	C14—C19	1.417 (5)
O4—C12	1.363 (4)	C15—C16	1.356 (6)
O4—B1	1.426 (5)	C15—H15	0.9300
O5—C22	1.324 (4)	C16—C17	1.399 (6)
O5—B1	1.502 (5)	C16—H16	0.9300
O6—C22	1.216 (4)	C17—C18	1.355 (6)
C1—C2	1.362 (5)	C17—H17	0.9300
C1—C10	1.404 (5)	C18—C19	1.417 (5)
C2—C3	1.408 (5)	C18—H18	0.9300
C2—H2	0.9300	C19—C20	1.400 (5)
C3—C4	1.412 (5)	C20—C21	1.368 (5)
C3—C8	1.417 (5)	C20—H20	0.9300
C4—C5	1.355 (6)	C21—C22	1.473 (5)
C4—H4	0.9300	N5—C24	1.466 (5)
C5—C6	1.395 (6)	N5—C23	1.467 (5)
C5—H5	0.9300	N5—H25A	1.0035
C6—C7	1.360 (5)	N5—H25B	0.9530
C6—H6	0.9300	C23—H23A	0.9600
C7—C8	1.412 (5)	C23—H23B	0.9600
C7—H7	0.9300	C23—H23C	0.9600
C8—C9	1.416 (5)	C24—H24A	0.9600
C9—C10	1.364 (5)	C24—H24B	0.9600
C9—H9	0.9300	C24—H24C	0.9600
C10—C11	1.482 (5)	O7—H27	0.85 (3)
C12—C13	1.361 (5)		
			
C1—O1—B1	116.9 (3)	C16—C15—H15	119.9
C11—O2—B1	122.2 (3)	C14—C15—H15	119.9
C12—O4—B1	116.5 (3)	C15—C16—C17	121.8 (5)
C22—O5—B1	121.6 (3)	C15—C16—H16	119.1
O1—C1—C2	120.0 (4)	C17—C16—H16	119.1
O1—C1—C10	119.9 (4)	C18—C17—C16	119.2 (5)
C2—C1—C10	120.1 (4)	C18—C17—H17	120.4
C1—C2—C3	120.5 (4)	C16—C17—H17	120.4
C1—C2—H2	119.7	C17—C18—C19	121.4 (5)
C3—C2—H2	119.7	C17—C18—H18	119.3
C2—C3—C4	121.9 (4)	C19—C18—H18	119.3
C2—C3—C8	120.2 (4)	C20—C19—C18	122.9 (4)
C4—C3—C8	117.9 (4)	C20—C19—C14	118.5 (4)
C5—C4—C3	121.0 (5)	C18—C19—C14	118.6 (4)
C5—C4—H4	119.5	C21—C20—C19	121.6 (4)
C3—C4—H4	119.5	C21—C20—H20	119.2
C4—C5—C6	120.9 (4)	C19—C20—H20	119.2
C4—C5—H5	119.5	C20—C21—C12	119.7 (4)
C6—C5—H5	119.5	C20—C21—C22	119.8 (4)
C7—C6—C5	120.2 (4)	C12—C21—C22	120.5 (4)
C7—C6—H6	119.9	O6—C22—O5	120.7 (4)
C5—C6—H6	119.9	O6—C22—C21	123.3 (4)
C6—C7—C8	120.4 (5)	O5—C22—C21	116.0 (4)
C6—C7—H7	119.8	O4—B1—O1	110.7 (4)
C8—C7—H7	119.8	O4—B1—O2	107.7 (3)
C7—C8—C9	123.0 (4)	O1—B1—O2	112.4 (3)
C7—C8—C3	119.6 (4)	O4—B1—O5	112.0 (3)
C9—C8—C3	117.4 (4)	O1—B1—O5	107.9 (3)
C10—C9—C8	121.4 (4)	O2—B1—O5	106.1 (4)
C10—C9—H9	119.3	C24—N5—C23	113.5 (4)
C8—C9—H9	119.3	C24—N5—H25A	115.4
C9—C10—C1	120.4 (4)	C23—N5—H25A	107.4
C9—C10—C11	119.6 (4)	C24—N5—H25B	109.4
C1—C10—C11	120.0 (4)	C23—N5—H25B	110.4
O3—C11—O2	119.9 (4)	H25A—N5—H25B	99.9
O3—C11—C10	123.9 (4)	N5—C23—H23A	109.5
O2—C11—C10	116.2 (4)	N5—C23—H23B	109.5
C13—C12—O4	120.1 (4)	H23A—C23—H23B	109.5
C13—C12—C21	120.1 (4)	N5—C23—H23C	109.5
O4—C12—C21	119.7 (4)	H23A—C23—H23C	109.5
C12—C13—C14	121.2 (4)	H23B—C23—H23C	109.5
C12—C13—H13	119.4	N5—C24—H24A	109.5
C14—C13—H13	119.4	N5—C24—H24B	109.5
C13—C14—C15	122.3 (4)	H24A—C24—H24B	109.5
C13—C14—C19	118.9 (4)	N5—C24—H24C	109.5
C15—C14—C19	118.8 (4)	H24A—C24—H24C	109.5
C16—C15—C14	120.2 (5)	H24B—C24—H24C	109.5
			
B1—O1—C1—C2	152.4 (4)	C13—C14—C15—C16	180.0 (4)
B1—O1—C1—C10	−28.0 (5)	C19—C14—C15—C16	−0.2 (7)
O1—C1—C2—C3	179.7 (4)	C14—C15—C16—C17	0.6 (8)
C10—C1—C2—C3	0.1 (6)	C15—C16—C17—C18	−0.4 (9)
C1—C2—C3—C4	178.0 (4)	C16—C17—C18—C19	−0.3 (8)
C1—C2—C3—C8	−0.6 (6)	C17—C18—C19—C20	179.8 (5)
C2—C3—C4—C5	−177.7 (4)	C17—C18—C19—C14	0.8 (7)
C8—C3—C4—C5	1.0 (6)	C13—C14—C19—C20	0.2 (6)
C3—C4—C5—C6	−0.7 (7)	C15—C14—C19—C20	−179.6 (4)
C4—C5—C6—C7	−0.2 (8)	C13—C14—C19—C18	179.3 (4)
C5—C6—C7—C8	0.8 (7)	C15—C14—C19—C18	−0.5 (6)
C6—C7—C8—C9	177.5 (4)	C18—C19—C20—C21	−177.6 (4)
C6—C7—C8—C3	−0.5 (6)	C14—C19—C20—C21	1.4 (6)
C2—C3—C8—C7	178.3 (4)	C19—C20—C21—C12	−1.7 (6)
C4—C3—C8—C7	−0.4 (6)	C19—C20—C21—C22	178.9 (4)
C2—C3—C8—C9	0.2 (6)	C13—C12—C21—C20	0.2 (6)
C4—C3—C8—C9	−178.5 (4)	O4—C12—C21—C20	−178.1 (3)
C7—C8—C9—C10	−177.3 (4)	C13—C12—C21—C22	179.6 (4)
C3—C8—C9—C10	0.7 (6)	O4—C12—C21—C22	1.4 (5)
C8—C9—C10—C1	−1.2 (6)	B1—O5—C22—O6	−171.3 (4)
C8—C9—C10—C11	178.9 (4)	B1—O5—C22—C21	8.7 (5)
O1—C1—C10—C9	−178.8 (4)	C20—C21—C22—O6	8.0 (6)
C2—C1—C10—C9	0.8 (6)	C12—C21—C22—O6	−171.4 (4)
O1—C1—C10—C11	1.1 (6)	C20—C21—C22—O5	−172.0 (4)
C2—C1—C10—C11	−179.3 (4)	C12—C21—C22—O5	8.5 (5)
B1—O2—C11—O3	−173.9 (4)	C12—O4—B1—O1	163.5 (3)
B1—O2—C11—C10	6.0 (6)	C12—O4—B1—O2	−73.2 (4)
C9—C10—C11—O3	9.7 (6)	C12—O4—B1—O5	43.1 (5)
C1—C10—C11—O3	−170.2 (4)	C1—O1—B1—O4	161.8 (3)
C9—C10—C11—O2	−170.2 (4)	C1—O1—B1—O2	41.4 (5)
C1—C10—C11—O2	9.9 (6)	C1—O1—B1—O5	−75.3 (4)
B1—O4—C12—C13	153.2 (4)	C11—O2—B1—O4	−153.3 (4)
B1—O4—C12—C21	−28.6 (5)	C11—O2—B1—O1	−31.1 (6)
O4—C12—C13—C14	179.7 (4)	C11—O2—B1—O5	86.6 (4)
C21—C12—C13—C14	1.5 (6)	C22—O5—B1—O4	−34.1 (5)
C12—C13—C14—C15	178.2 (4)	C22—O5—B1—O1	−156.1 (3)
C12—C13—C14—C19	−1.7 (6)	C22—O5—B1—O2	83.2 (4)

### Hydrogen-bond geometry (Å, °)

**Table:**

*D*—H···*A*	*D*—H	H···*A*	*D*···*A*	*D*—H···*A*
N5—H25A···O3^i^	1.00	1.88	2.849 (4)	162
N5—H25B···O6^ii^	0.95	2.12	2.839 (4)	131
N5—H25B···O3^iii^	0.95	2.33	2.871 (4)	116
O7—H27···O5	0.85 (3)	2.18 (4)	3.010 (3)	163.3 (12)

Symmetry codes: (i) *x*, −*y*+1, *z*−1/2; (ii) *x*, *y*+1, *z*; (iii) −*x*, −*y*+1, −*z*+1.
